# Identifying Genome-Wide Sequence Variations and Candidate Genes Implicated in Self-Incompatibility by Resequencing *Fragaria viridis*

**DOI:** 10.3390/ijms20051039

**Published:** 2019-02-27

**Authors:** Jianke Du, Yan Lv, Jinsong Xiong, Chunfeng Ge, Shahid Iqbal, Yushan Qiao

**Affiliations:** 1 Laboratory of Fruit Biotechnology, College of Horticulture, Nanjing Agricultural University, Nanjing 210095, Jiangsu, China; 2017204014@njau.edu.cn (J.D.); 2017104019@njau.edu.cn (Y.L.); jsxiong@njau.edu.cn (J.X.); 2017204045@njau.edu.cn (S.I.); 2 Institute of Botany, Jiangsu Province and Chinese Academy of Sciences, Nanjing 210014, Jiangsu, China; 18761866172@163.com

**Keywords:** *Fragaria viridis*, genome, variations, resequencing, self-incompatibility, *FIP2-like*

## Abstract

It is clear that the incompatibility system in *Fragaria* is gametophytic, however, the genetic mechanism behind this remains elusive. Eleven second-generation lines of *Fragaria viridis* with different compatibility were obtained by manual self-pollination, which can be displayed directly by the level of fruit-set rate. We sequenced two second-generation selfing lines with large differences in fruit-set rate: Ls-S_2_-53 as a self-incompatible sequencing sample, and Ls-S_2_-76 as a strong self-compatible sequencing sample. *Fragaria vesca* was used as a completely self-compatible reference sample, and the genome-wide variations were identified and subsequently annotated. The distribution of polymorphisms is similar on each chromosome between the two sequencing samples, however, the distribution regions and the number of homozygous variations are inconsistent. Expression pattern analysis showed that six candidate genes were significantly associated with self-incompatibility. Using *F. vesca* as a reference, we focused our attention on the gene *FIP2-like* (FH protein interacting protein), associated with actin cytoskeleton formation, as the resulting proteins in Ls-S_2_-53 and Ls-S_2_-76 have each lost a number of different amino acids. Suppression of *FIP2-like* to some extent inhibits germination of pollen grains and growth of pollen tubes by reducing F-actin of the pollen tube tips. Our results suggest that the differential distribution of homozygous variations affects *F. viridis* fruit-set rate and that the fully encoded FIP2-like can function normally to promote F-actin formation, while the new FIP2-like proteins with shortened amino acid sequences have influenced the (in)compatibility of two selfing lines of *F. viridis*.

## 1. Introduction

*Fragaria viridis* Weston, known as the green strawberry, belongs to a diploid wild resource of the genus *Fragaria* of Rosaceae and is a potential breeding material for the character optimization of cultivated strawberry. It has a variety of excellent properties, such as firm flesh, remontant flowering habit, good flower characteristics, and an acidic apple-like aroma [1]. Although self-compatibility has been reported in *Fragaria vesca* [2], *F. viridis* has gametophytic self-incompatibility [1,3]. In contrast to the genera *Malus*, *Pyrus*, and *Prunus* [4], *F. viridis* has two controlling sites; thus, the self-incompatibility mechanisms that control the pollen–pistil interaction are difficult to understand [4,5]. Based on manually self-pollinated first generation Ls-S_1_-2 of *F. viridis* 42 [6], a group of *F. viridis* second-generation selfing lines with large differences in fruit-set rate was obtained by manual self-pollination. The lowest fruit-set rate was only 2%, while the highest was up to 80%, however, this is still different from the completely compatible *F. vesca* 41 (100% rate of fruit-set in this study), and the molecular mechanisms governing the differences in fruit-set rate are unknown.

Currently, over 14 species of fruit tree genome sketches have been released, including five species of Rosaceae: apple [7], pear [8], strawberry [9], peach [10], and Japanese apricot [11]. Shulaev et al. [9] published a sketch of diploid *F. vesca* genome and comprehensively annotated the gene function. The genome consists of seven chromosomes of about 230 Mb, which lack the analogous repeat sequences that exist in other Rosaceae species [9]. Over several years, scientists further improved the genomic map and annotation information of *F. vesca* [12,13,14,15]. The release of whole genomes of strawberry promotes the scientific understanding of the chromosomal composition of octoploid strawberries [16], functional genomics [13,17], interspecies collinearity [9], the construction of high-precision maps, QTL mapping analysis [18], and molecular breeding technology [18,19].

With the completion of whole-genome sequencing of many species and the rapid development of high-throughput sequencing and resequencing technology, significant progress has been made in genetic evolution [20], molecular markers [21,22], transposon analysis [23], and genetic prediction of important agronomic traits [20,21,24]. Resequencing samples with significant differences and combining with bioinformatic analysis can allow the rapid investigation of genetic differences across genomes and the prediction of candidate genes for important agronomic traits, such as apple flowering genes [25] and grape early maturing genes [26]. This strategy has been used to investigate the genetic variation and to find the candidate genes related to agronomic traits among different samples and has also been applied successfully on plants of the genus *Fragaria* [27]. 

Many fruit trees of the family Rosaceae possess an S-RNase-based gametophytic self-incompatibility (GSI) system [4,28]. This involves a complex genetic mechanism, and the display of the complete trait of self-incompatibility not only depends on the S-site genes (pistil S gene and pollen S gene) but, also, several modifier genes associated with complex mechanisms that have been gradually explored [29]. These modifier genes are involved in multiple signal transduction systems [29], for example, calcium ion signaling [29,30,31], reactive oxygen signaling [32], hormone signaling [33], inositol phosphate signaling [34,35], biochemical metabolism processes [36], programmed cell death (PCD) [32,37], and cell wall and cytoskeletal construction [28,38,39,40,41]. These modifiers, distributed in a determined location of the chromosome, will affect the intensity of self-incompatibility to varying degrees, and be directly reflected in the fruit-set rate. Therefore, to enable a deep understanding of the self-incompatibility of green strawberry, it is important that the related genes are mined by whole-genome sequencing technology.

The hypothesis of this work is that the functional change of the modifier genes caused by sequence variations leads to differences in fruit-set rate. Using *F. vesca* as the reference genome [9], two green strawberry second-generation selfing lines with large differences in fruit-set rate were selected as sequencing samples, namely, Ls-S_2_-53 as a self-incompatible line, and Ls-S_2_-76 as a strong self-compatible line. By comparing the two samples with the reference genome, many genetic variations and molecular markers related to agronomic traits were discovered. The series of genes related to self-incompatibility was further predicted by analyzing the variations that may influence gene function, which has provided some clues for modifiers research on the GSI of plants of the genus *Fragaria*. The sequence and function of a gene (FH protein interacting protein, *FIP2-like*), which may be related to F-actin formation, was verified, and its role in the samples that have different degrees of self-(in)compatibility was further analyzed.

## 2. Results

### 2.1. Traits of Fruit and Pollen Tubes in Styles

**Figure 1:**
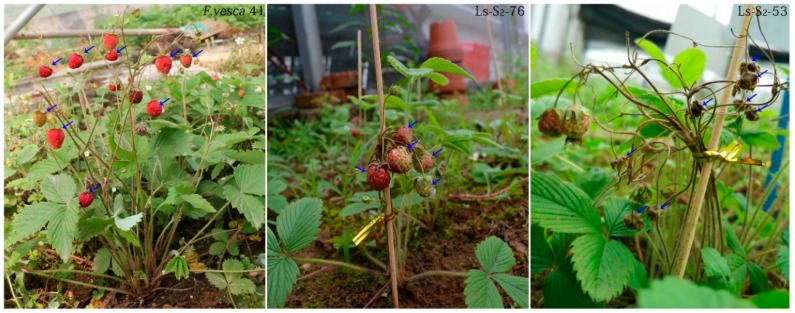
The field performances of *F. vesca* 41, Ls-S_2_-53, and Ls-S_2_-76. Arrows mark the growing condition of fruit for 25 days after manual self-pollination.

**Figure 2:**
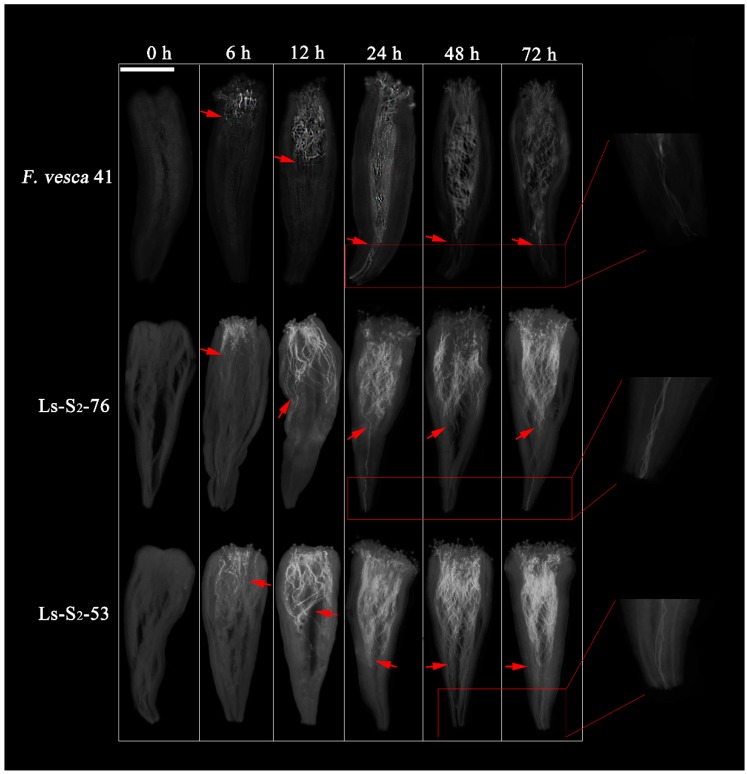
Results of dynamic observation of pollen tube growth in styles. Times of 0, 6, 12, 24, 48, and 72 h each indicate the growth state of the pollen tubes of *F. vesca* 41, Ls-S_2_-76, and Ls-S_2_-53 after manual self-pollination. The locations indicated by red arrows are where most of the pollen tubes arrived. The red squares indicate the details of the style basis passed by pollen tubes at different time in different samples. Scale bar = 0.5 mm. The growth rate of the pollen tubes was similar within 12 h between the three different samples, and reached about 1/3 of the style at 12 h. Most pollen tubes of *F. vesca* reached the base of the styles, while those of two green strawberry lines reached 2/3 of styles at 24 h. Unlike the Ls-S_2_-53 lines, some styles of Ls-S_2_-76 were crossed by at least one pollen tube at 24 h.

The fruit-set rate of the 13 green strawberry lines significantly differed by between 2% and 80% (Table 1). The fruit-set rates of Ls-S_2_-30 and Ls-S_2_-76 were higher than other green strawberry lines, being 80% and 75%, respectively (Figure 1; Table 1). The difference of fruit-set rate between both was not obvious, however, the fruit malformation rate of Ls-S_2_-30 is markedly higher than Ls-S_2_-76. Therefore, we chose Ls-S_2_-76 as the intensive compatibility sequencing sample and selected the Ls-S_2_-53 with the lowest fruit-set rate as the incompatibility sequencing sample. *F. vesca* shows a very high fruit-set rate (Table 1) and is an excellent reference sample of complete compatibility [3,9]. The three differential trait samples provided the material basis for the analysis of sequence polymorphism, structural variation, and mining of self-incompatibility-related genes. The dynamic observation of pollen tube growth in the styles showed that the growth rate of pollen tubes of *F. vesca* was faster than for the two green strawberry lines from 12 to 24 h after manual self-pollination (Figure 2); there was a significant difference in the proportion of the styles crossed by pollen tubes at 24 h: for *F. vesca*, Ls-S_2_-76, and Ls-S_2_-53, this proportion was 80%, 12%, and 0%, respectively (Figure 2; Table 2). All styles of *F. vesca* 41 were penetrated by at least one pollen tube at 48 h; Ls-S_2_-76 increased to 90% while Ls-S_2_-53 accounted for only 6%. At 72 h, the number of styles crossed by pollen tube did not show an increase in either Ls-S_2_-53 or Ls-S_2_-76 (Figure 2; Table 2).

### 2.2. Genome Sequencing and Mapping of Reads to the Reference

Whole genome sequencing (WGS) produced 139,726,788 read pairs, 72,849,376 for Ls-S_2_-53, and 66,877,412 for Ls-S_2_-76. After pre-processing, 122,329,816 read pairs were retained, 63,518,251 read pairs from Ls-S_2_-53 and 58,811,565 read pairs from Ls-S_2_-76, and were finally mapped to the *F. vesca* reference genome, with an average depth of 80× and 75×, respectively (Table 3). Above 90% (coverage depth ≥1×) of the reference genomic regions were covered by either of two samples with bases. The percentage of sequences (Q30) reached more than 91% and 39% GC content in both of the two sequencing samples (Table 3). More details of the original sequencing data quality, including base type distribution, base quality distribution, sequencing depth distribution, insert size distribution, and principal coordinate analysis of two samples are presented in Figure S1. The above sequencing results provide a reliable database for the detection of sample polymorphisms, and the data was submitted to the National Center for Biotechnology Information (NCBI) Sequence Read Archive (SRA) database. The accession number was PRJNA510489.

### 2.3. Detection and Characteristics of Single-Nucleotide Polymorphisms (SNPs), Structural Variations (SVs), and Indels

Using *F. vesca* as a reference, 2,721,415 single-nucleotide polymorphisms (SNPs), 409,626 indels, and 32,534 structural variations (SVs) were identified in the Ls-S_2_-53 genome; 84.15% of SNPs and 83.98% of indels were homozygotes. Meanwhile, 2,718,829 SNPs, 409,159 indels, and 31,815 SVs were identified in the Ls-S_2_-76 genome; 84.25% of SNPs and 84.02% of indels were homozygotes (Additional File 1). A total of 12,867.2 and 12,855.0 SNPs per Mb, 1936.8 and 1934.6 indels per Mb, and 153.8 and 150.4 SVs per Mb were detected on chromosomes of Ls-S_2_-53 and Ls-S_2_-76, respectively (Additional File 1). All of the identified genetic variations involved 16,245 genes. Additionally, a total of 190,729 SNPs and 33,488 indels were identified between the Ls-S_2_-53 genome and the Ls-S_2_-76 genome according to whether variations on the same locus in the two sequencing samples were consistent or not (Additional File 1).

Based on the alignment results between samples and the reference genome, we further detailed and counted the variations. A total of 73,775 and 73,923 synonymous (syn) SNPs and 86,025 and 85,928 nonsynonymous (nonsyn) SNPs, which result in amino acid changes, were located in Ls-S_2_-53 and Ls-S_2_-76 coding sequences (CDSs), respectively. Depending on the substitution of nucleotides, SNPs can be divided into transitions (C/T and G/A) and transversions (C/A, G/C, A/T, and T/G); the ratio of transitions to transversions (Ts/Tv) was 1.57 in both of the two sequencing samples. We counted the numbers of indels with different sizes in the whole genome and the CDS regions, and further analysis demonstrates that the majority of insertions and deletions were in the range of 1–4 bp in both sequencing samples (Figure S2). A total of 74,998 and 75,012 insertions (INSs) and 70,858 and 70,702 deletions (DELs) in the genomic region, and 3325 and 3332 INSs, and 3399 and 3381 DELs in the CDS regions, are distributed in Ls-S_2_-53 and Ls-S_2_-76, respectively. There was no significant difference in the proportion of each SV between Ls-S_2_-53 and Ls-S_2_-76; Ls-S_2_-53 contains 32,534 SVs compared to the reference genome, including 17,099 (52%) DELs, 7366 (23%) interchromosomal translocations (CTXs), 2473 (8%) intrachromosomal translocations (ITXs), 1809 (5%) inversions (INVs), 3785 (11%) unknown SVs, and two INSs, however, no INSs were found on Ls-S_2_-76. Interestingly, the DEL, CTX, and ITX annotations show large majority were found in both of the two sequencing samples (Figure S3).

### 2.4. Distribution of SNPs, Indels, and SVs

**Figure 3:**
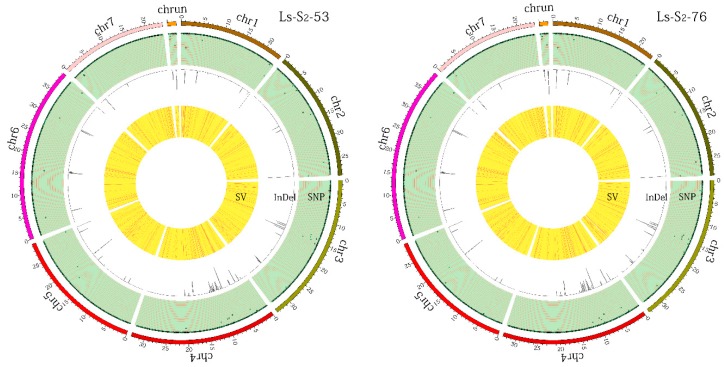
The distribution of DNA polymorphisms (single-nucleotide polymorphisms (SNPs), indels, structural variations (SVs)) in *F. viridis* resequencing samples Ls-S_2_-53 and Ls-S_2_-76, compared with the reference sample genome (*F. vesca*). The three rings, from outside to inside, represent all detected SNPs, indels, and SVs, respectively. The total variation distribution in the two sequencing samples is highly similar.

**Figure 4:**
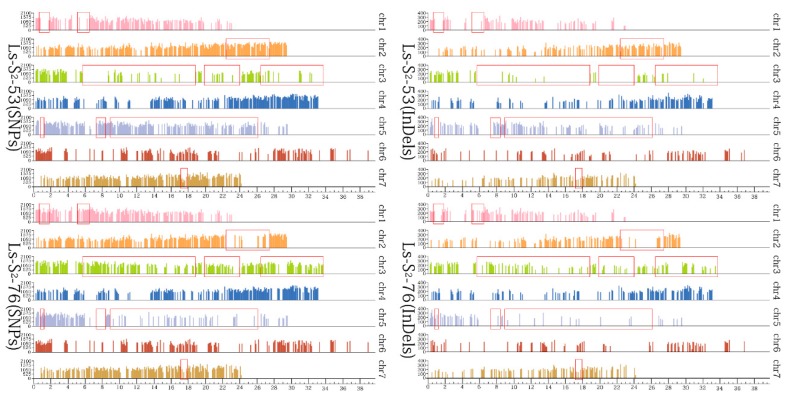
The specific distribution area of homozygous mutations (SNPs, indels) in Ls-S_2_-53 and Ls-S_2_-76 using a sliding window of 100 K with heterozygosity (<10%) as the parameter. The axis of abscissas and ordinates indicate the chromosomal location and number of variations, respectively. The different distribution areas of homozygous mutations were marked by the red rectangles and mainly distributed on chromosomes 2, 3, and 5.

The detailed distribution of SNPs, indels, and SVs of the two sequencing samples (Ls-S_2_-53 and Ls-S_2_-76) were identified compared with the *F. vesca* reference genome, and as shown in Figure 3 and Additional File 1. The distribution of polymorphisms is similar in Ls-S_2_-53 and Ls-S_2_-76 but, however, is uneven within chromosomes as well as between each chromosome within Ls-S_2_-53 or Ls-S_2_-76. The largest numbers of 519,100 and 518,521 SNPs, and 79,658 and 79,531 indels, were observed on chromosome 6, while the lowest numbers of 302,695 and 300,168 SNPs, and 45,618 and 45,113 indels, were observed on chromosome 1, in Ls-S_2_-53 and Ls-S_2_-76, respectively (Additional File 1). Other chromosomes showed similar results. The distribution of homozygous variations was significantly different in Ls-S_2_-53 and Ls-S_2_-76 compared with the *F. vesca* reference genome (Additional File 1). In Ls-S_2_-53, the largest proportion of homozygous SNPs was observed on chromosome 2, while the lowest proportion was observed on chromosome 3; in Ls-S_2_-76, the largest proportion of homozygous SNPs was observed on chromosome 7, while the lowest proportion was observed on chromosome 6. Compared with Ls-S_2_-76, Ls-S_2_-53 had a significantly higher proportion of homozygosity on chromosomes 2, 4, 5, and 7, and vice versa, for chromosomes 1, 3, and 6. Using a sliding window of 100 K with heterozygosity (<10%) as the parameter, a different distribution area of homozygous mutations was found; 10 abundant SNPs were detected on a high region on chromosome 1 (0.7–1.8 Mb, 5.1–6.5 Mb), chromosome 2 (22.3–27.5 Mb), chromosome 3 (5.7–18.8 Mb, 19.8–24.0 Mb, 26.4–33.7 Mb), chromosome 5 (0.7–1.2 Mb, 7.3–8.4 Mb, 8.9–26.1 Mb), and chromosome 7 (17.1–17.6 Mb) between Ls-S_2_-53 and Ls-S_2_-76 (Figure 4). Similar areas of difference were also represented on indels.

**Figure 5:**
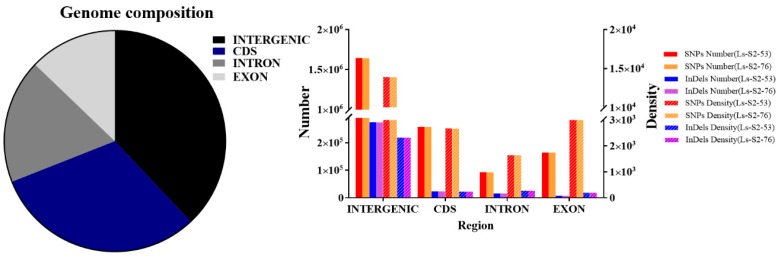
The chromosome composition and variation distribution of SNPs and indels in different regions of the *F. viridis* genome.

We calculated the size of genome composition involving intergenic regions, genic regions, exon regions, and intron regions (Figure 5). The intergenic regions were significantly longer than genic regions, and the number of variations (SNPs and indels) in intergenic regions was larger. The variation distribution density of SNPs and indels was further analyzed in different regions of the genome. The SNPs’ distribution density order, from high to low, was intergenic regions, exon regions, genic regions, and intron regions, while the indels’ distribution density order, from high to low, was intergenic regions, intron regions, genic regions, and exon regions.

### 2.5. The Annotation and Function Analysis of SNPs, Indels, and SVs

**Figure 6:**
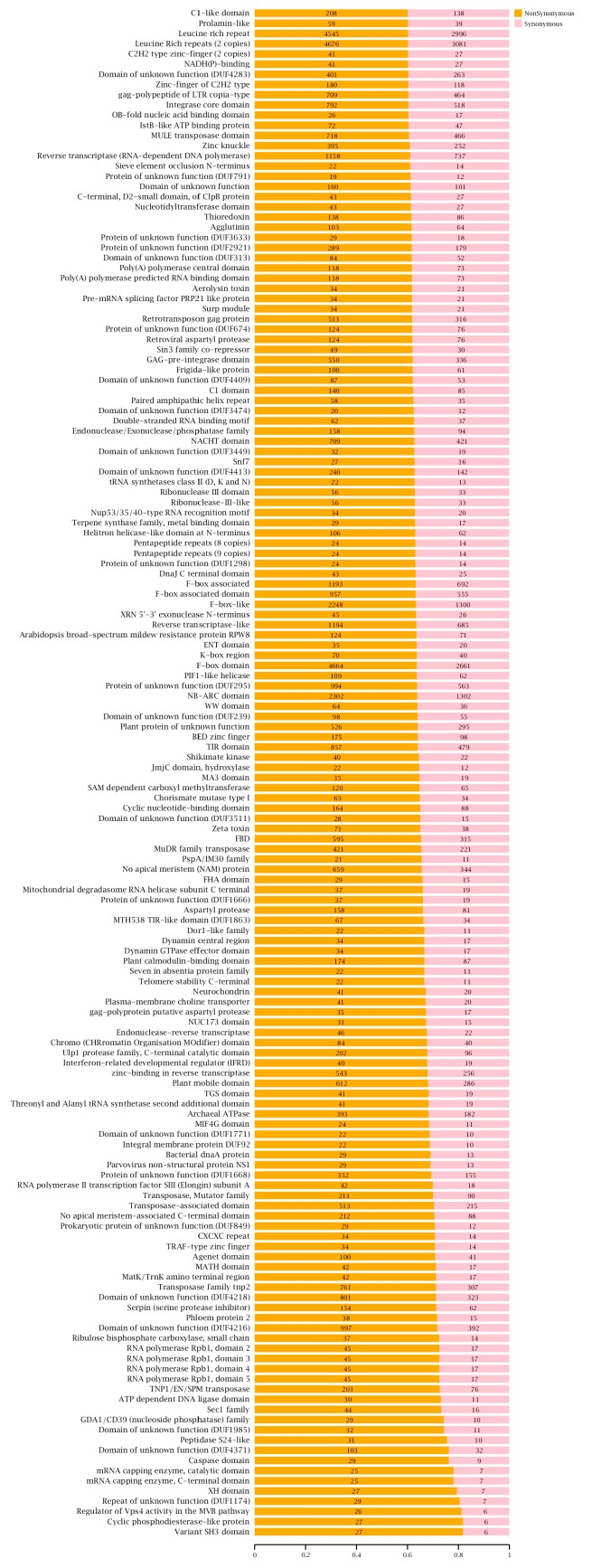
The distribution information of nonsynonymous (nonsyn) and synonymous (syn) SNPs in different Pfam-containing genes in *Fragaria* genomes. The Pfam-containing gene families with more than 30 nonsyn and syn SNPs (ratio of nonsyn to syn SNPs > 0.6) were analyzed and listed. The chi^2^ significance of the involved nonsyn and syn SNP distributions in each Pfam group was evaluated, *p*-value < 0.001.

SNPs, although containing small differences, have a great impact on the function of genes and biological traits, especially those distributed in the Pfam-containing genes; the ratio of nonsyn to syn SNPs is higher in these genes which indicating that the Pfam domains (Function domain of the gene) possibly had more amino acid substitutions, and the probability of functional changes is higher [25,42]. Therefore, the distribution information of the SNPs in Pfam-containing genes was further analyzed (Figure 6). The result shows that more SNPs and a higher ratio of nonsyn to syn SNPs were found in leucine-rich repeats, F-box domain, NB-ARC domain, thioredoxin, plant calmodulin-binding domain, NADH(P)-binding, and pentapeptide repeats, indicating that the genes containing these Pfam domains may have changes of function. The variations result in amino acid changes, such as nonsyn SNP mutations, frame-shifts, SVs in coding sequence associated with the genes in Ls-S_2_-53 and Ls-S_2_-76, details of which can be found in Additional File 2. The same type of genetic variation between Ls-S_2_-53 genome and Ls-S_2_-76 genome was also identified (Additional File 3). Furthermore, these genes may have amino acid changes; the detailed functional annotation data from Swiss-Prot, NR, COG, GO, and KEGG databases, in Ls-S_2_-53 and Ls-S_2_-76, can be found in Additional File 4.

In total, 4794 genes (Additional File 2) were assigned gene ontology (GO) annotations by GO analysis, as shown in Figure S4. All the genes (Additional File 2) that exhibit SNP mutations, indels or SVs were classified into three categories, namely, cellular component, molecular function, and biological process. Cell part (1700 genes, 35.46%), cell (1695 genes, 35.36%), and organelle (1323 genes, 27.60%) dominated in the cellular component category. With respect to the molecular function, genes were associated with catalytic activity (2416 genes, 50.40%), binding (2227 genes, 46.45%), and transporter activity (199 genes, 4.15%). Genes associated with the metabolic process (2791 genes, 58.22%), cellular process (2176 genes, 45.39%), and biological regulation (809 genes, 16.88%) were dominant in the biological process category. Meanwhile, these genes were also assigned to KEGG annotations analysis (Additional File 5, Figure S5). A total of 98 KEGG pathways were involved. A total of 12 KEGG pathways were significantly enriched, including plant hormone signal transduction (ko04075), spliceosome (ko03040), ribosome (ko03010), plant–pathogen interaction (ko04626), ubiquitin-mediated proteolysis (ko04120), and RNA degradation (ko03018; *p*-value < 0.1 and FDR < 0.1).

**Figure 7:**
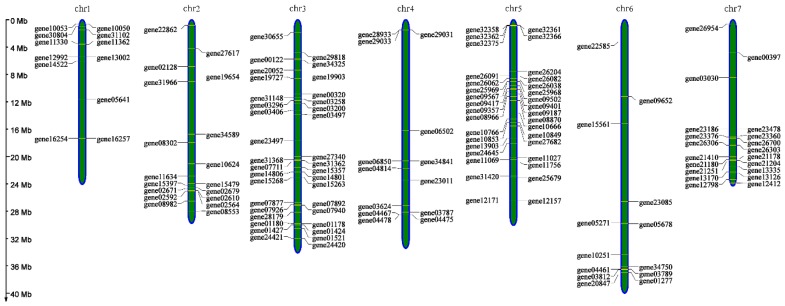
Constructed physical maps of *Fragaria* using 146 predicted genes related to self-incompatibility. The IDs for the genes are shown on the chromosome and the chromosomal locations of genes are indicated by the axis of ordinates on the right.

According to Additional File 2, all of the different genes that might be involved in incompatibility traits between three strawberry samples were identified, as shown in Additional File 6. Subsequently, 146 identified genes (Additional File 7) were used to construct physical maps, which were screened out in the same way as for Additional File 3. The details of these predicted incompatibility genes, including locations and distributions, are shown in Figure 7. These genes were obtained based on previous research in other species and combined with an amino acid change of the genes between the two sequencing samples. A total of 34 and 36 predicted incompatibility genes were located on chromosomes 5 and 3, respectively, while just 12 and 13 were located on chromosomes 1 and 6, respectively. It is interesting that the predicted incompatibility genes are more likely to be located on chromosomes which contain more different distribution areas of homozygous mutations between Ls-S_2_-53 and Ls-S_2_-76, such as chromosomes 3 and 5.

### 2.6. RT-qPCR Analysis

**Figure 8:**
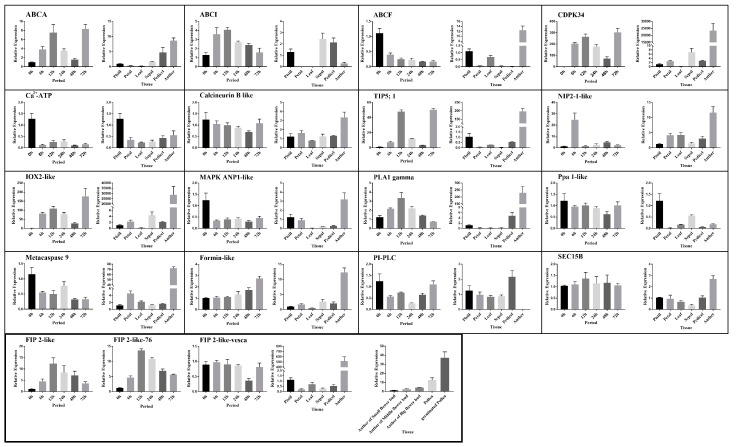
Relative gene expression of selected genes in manually self-pollinated pistil after 0, 6, 12, 24, 48, and 72 h, and tissue-specific (style, leaf, petal, sepal, pedicel, anther) expression pattern analysis. The materials used in FIP2-like-76 and FIP2-like-vesca are, respectively, the pistil of Ls-S_2_-76 and *F. vesca* 41, and the others are from Ls-S_2_-53. Mean values and standard deviations (SD) were calculated from three replicates, *p* < 0.05. The IDs and annotation information of these abbreviated genes are displayed in Table S1 and Additional File 6.

In order to verify the role of the selected differential candidate genes in self-incompatibility, we randomly selected 17 genes for quantitative expression analysis (Figure 8). The expression levels of all genes in different periods after manual self-pollination in the incompatible samples (Ls-S_2_-53) were analyzed. It was found that *ABCA* (ABC transporter A family member), *ABCI* (ABC transporter I family member), *CDPK34* (calcium-dependent protein kinase 34), *TIP5;1* (tonoplast membrane intrinsic protein), *NIP2-1-like* (nodulin 26-like intrinsic protein), *IOX2-like* (inositol oxygenase), *PLA1Ⅱgamma* (phospholipase A1-IIgamma-like), *FIP2-like*, and *SEC15B* (exocyst complex component SEC15B) present high expression levels after pollination; within 48 h after pollination, *ABCA*, *ABCI*, *CDPK34*, *TIP5;1*, *NIP2-1-like*, *IOX2-like*, *PLA1Ⅱgamma*, and *FIP2-like* showed a trend of rising first and then decreasing. It is unusual that the expression of *ABCA*, *CDPK34*, *TIP5;1*, and *IOX2-like* suddenly rose again 72 h after pollination. *ABCF* (ABC transporter F family member), *Ca^2+^-ATPase* (calcium-transporting ATPase), *Calcineurin B* (calcineurin B-like protein), *MAPK ANP1-like* (mitogen-activated protein kinase ANP1-like), *Ppa 1-like* (inorganic pyrophosphatase), *Metacaspase 9*, and *PI-PLC* (PI-PLC X-box domain-containing protein) decreased after pollination. Tissue-specific expression analysis revealed that 14 genes were highly expressed in anthers; seven of these genes (*ABCF*, *CDPK34*, *TIP5;1*, *IOX2-like*, *PLA1Ⅱgamma*, *FIP2-like*, *Metacaspase 9*) were specifically expressed in anthers, and the expression level of the other three genes was slightly higher in other floral organs, such as pistils and peduncles, and 15 genes were higher in pistils than in leaves. Our results indicate that these genes were clearly expressed in flower organs, especially in anthers and pistils. The expression level of six genes increase significantly and have a similar trend to *S-RNase* after pollination [43], which implies that these genes may have an effect on the incompatibility of *F. viridis*. To further analyze the role of *FIP2-like* candidate genes in self-incompatibility, we additionally analyzed the expression of *FIP2-like* in Ls-S_2_-76 and *F. vesca* samples according to the period described above. Ls-S_2_-53 was similar to Ls-S_2_-76 but was not consistent with *F. vesca* in expression patterns. The expression of *FIP2-like* in the different developmental stages of anthers was inconsistent and showed a tendency of gradual increase with the maturity of anthers, and was also higher in pollen than anthers.

### 2.7. Cloning and Sequence Analysis of FIP2-Like

**Figure 9:**
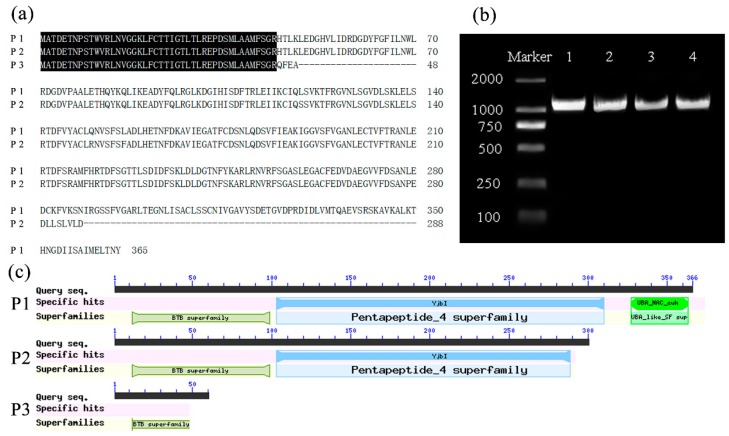
Sequence analysis of FIP2-like (*FIP2-like*). (**a**) Amino acid sequence alignment of FIP2-like of *F. vesca* 41 and Ls-S_2_-76 harbor P1 and P3, respectively, and Ls-S_2_-53 which harbors P2 and P3. (**b**) The full length of the *FIP2-like* gene. 1, 2 are obtained using the DNA and anther cDNA of *F. vesca* 41 as templates, respectively; 3 and 4 are respectively obtained using Ls-S_2_-53 and Ls-S_2_-76 anther cDNA as templates. (**c**) Contained structural domain of FIP2-like from P1, P2, and P3.

The *FIP2-like* gene was cloned from the cDNA and DNA of *F. vesca* 41 anthers as a template. The results of the alignment analysis showed that the gene had no intron (Figure S6), and the full-length sequence of CDS (Seq1) is 1098 bp (Figure 9b and Figure S6). *FIP2-like* is located in a different distribution area on chromosome 3, which demonstrates that the gene mainly contains heterozygous and homozygous variations in Ls-S_2_-53 and Ls-S_2_-76, respectively (Figure 4 and Figure 7). Using Ls-S_2_-53 and Ls-S_2_-76 cDNA as a template, a single sequence (Seq3) was obtained from Ls-S_2_-76, and 14 SNPs and a 2 bp DEL were found compared with *F. vesca* (Table 4). Additionally, the sequences Seq2 and Seq3 (Figure 9b and Figure S6) were obtained from Ls-S_2_-53, and 16 SNPs and a 23 bp DEL were found in Seq2 compared with *F. vesca*. The sequence information obtained by monoclonal sequencing was mainly consistent with the results of resequencing, which suggests that the resequencing data are reliable. The *FIP2-like* gene of *F. vesca* can be translated the sequence containing 365 amino acids (Figure 9a), including three domains (Figure 9c); the amino acid translation process of Seq2 and Seq3 terminated prematurely (Figure 9a and Figure S6) since the stop codon was obtained in advance, which was caused by FRAME_SHIFT at different sites of *FIP2-like* with the *F. vesca* as the reference. P2 lacks 77 amino acid residues, the terminal domain has been lost the entire functional area, and the pentapeptide amino acid functional domain was partially damaged; P3 lacks 317 amino acid residues and two domains were deleted completely (Figure 9a,c).

### 2.8. Transient Expression Assay

**Figure 10:**
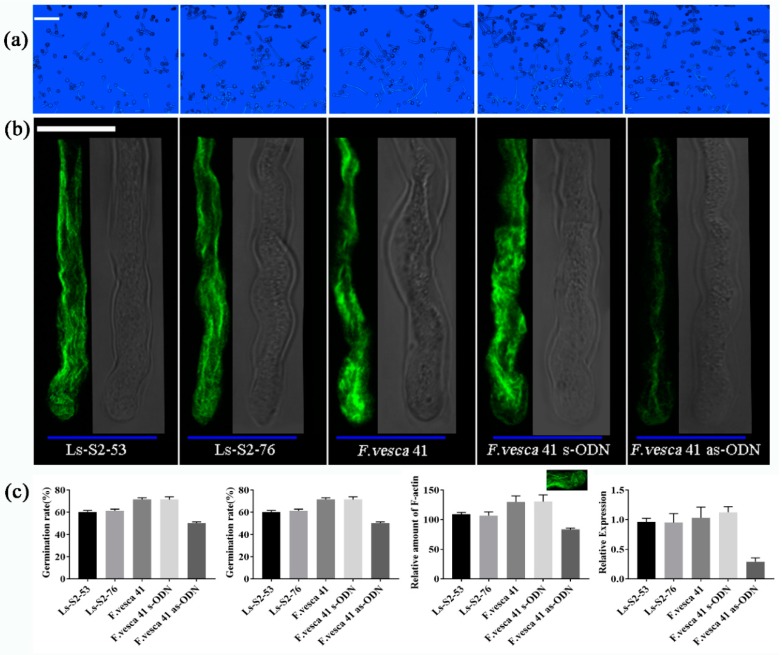
Pollen grain germination, pollen tube growth, and alterations of F-actin amount after antisense oligonucleotide (as-ODN) treatment. (**a**) Pollen grain germination and pollen tube growth for Ls-S_2_-53, Ls-S_2_-76, *F. vesca* 41, and *F. vesca* 41 of s-ODN treatment and *F. vesca* 41 of as-ODN treatment is displayed from left to right, respectively. Scale bar = 100 µm. (**b**) The amount of F-actin of pollen tubes, where the green fluorescence indicates F-actin filaments and the gray area corresponds to the bright field. The F-actin intensity of pollen tube tip was measured and further analyzed with the ImageJ software using the processed images (subtraction of background). Scale bar = 10 µm. (**c**) Pollen grain germination rate, average length of pollen tube, the F-actin density of pollen tube tip, and the relative expression of *FIP2-like* for the different samples and under the as-ODN treatments. Mean values and standard deviations (SD) were calculated from three replicates, *p* < 0.05.

The pollen grain germination rates of Ls-S_2_-53, Ls-S_2_-76, and *F. vesca* were calculated as 60.24%, 61.25%, and 71.49%, respectively (Figure 10a,c). *F. vesca* has a higher pollen grain germination rate than the two selfing lines of *F. viridis*, however, there was no significant difference between Ls-S_2_-76 and Ls-S_2_-53. Furthermore, the density of F-actin was investigated in three sample pollen tube tips by analyzing fluorescence intensity (Int/mm^2^). The fluorescence intensity between the two green strawberries Ls-S_2_-76 and Ls-S_2_-53 is similar and is lower than *F. vesca* (Figure 10b,c). The expression level of this gene decreased when adding antisense oligonucleotides (as-ODN) to medium compared with the control (s-ODN) (Figure 10c). When *FIP2-like* was suppressed by as-ODN in pollen, the germination rates and mean length of the pollen tubes were lower than in those treated with s-ODN as control (Figure 10a,c). The F-actin density of pollen tube tip was also further analyzed in *F. vesca*, after as-ODN treatment, and the accumulated fluorescence intensity lower than the control (s-ODN) (Figure 10b,c). These results indicate that suppression of *FIP2-like* to some extent inhibits the germination of pollen grains and the growth of the pollen tubes, and this is probably achieved by decreasing the formation of F-actin of the pollen cells. Additionally, the gene of *FIP2-like-GFP* with 35S promoter was transformed into *Nicotiana benthamiana*. It is clear that FIP2-like is localized on the cell membrane and nucleus (Figure S7).

## 3. Discussion

### 3.1. Resequencing Plays an Important Role in the Study of Genetic Variations

With the development of high-throughput sequencing technology and the release of genomic sketches of more species, the resequencing technique has become a relatively mature technology to study genetic differences such as SNPs and indels, which have been widely applied in marker-assisted and genomic selection, QTL mapping, positional cloning, and haplotype and pedigree analysis [21,22,23,24]. We resequenced two samples with large differences in fruit-set rate; a large number of mutations (SNPs, indels, and SVs) were identified, and it was further found that the distribution regions and number of homozygous variations were distinct between Ls-S_2_-53 and Ls-S_2_-76. We also obtained some candidate genes related to the self-incompatibility of *F. viridis* according to the sequence variations that might lead to changes in the encoded amino acids. The resequencing technique is a powerful tool and plays a significant role in rapidly identifying mutations, studying polymorphisms, and gene mining of important agronomic traits.

The ratio of nonsynonymous to synonymous (nonsyn/syn) SNPs is similar between Ls-S_2_-53 (1.17) and Ls-S_2_-76 (1.16). SNPs of nonsynonymous SNPs show a higher number than synonymous SNPs and are similar to peach [24], soybean [44], and rice [45]. These Pfam-containing genes with higher proportions of nonsynonymous SNPs may hold more functional diversifications of corresponding proteins. Large amounts of nonsynonymous SNPs, but few indels in the coding sequence, were found in Ls-S_2_-53 and Ls-S_2_-76; similar results were also observed in previous studies [25,46]. A higher ratio of Ti/Tv of the SNPs [24,42], single-based indels, account for the greatest proportion of indels in the genome and CDS than other types of indels [42], and this was also found in the two sequencing samples. The abovementioned regularity of several genetic variations was formed in the process of plant evolution to adapt to environments for survival.

The genetic relationships between species can be evaluated by analyzing the genetic variation [24,25,42]; the closer the genetic relationship is, the smaller the genetic differences are [42]. Compared with the reference genomes, there are more SNPs in the two samples, however, the number of SNPs between these two samples is extremely small as Ls-S_2_-53 and Ls-S_2_-76 are derived from same parent (Ls-S_1_-2). There are about 24 species of wild strawberry in the world, and ploidy diversity and the evolution of polyploid germplasm genomes is complex [47]. Genetic variation can be used not only to explore the genetic relationship of wild strawberries but also for genome component analysis and to deepen the understanding of polyploid origin through the development of molecular markers [16,19,48,49]. The certified genetic variation through genomic resequencing provides basic research data to explore the origin of the *Fragaria* polyploids.

The SNP variations in the intergenic regions, genic regions, coding regions, and introns are not uniform. The distribution of SNP variations of intergenic regions is the highest, however, the variations in other regions are relatively small. This phenomenon has also been shown in other species [24,25,42,50]. The reason for the large SNP variation in intergenic regions may be due to the large size of the region [25]; this view is supported by the fact that the size of different regions is directly proportional to the number of variations (Figure 5). Due to the existence of different DNA damage repair mechanisms, the repairability of chromosomes in different regions is different, which directly leads to the discrepancy in the number of the variations within the unit length of specific regions [51,52]. The genic regions have a smaller distribution density than intergenic regions in two resequencing samples, which implies a stronger DNA repair capacity in genic regions [51].

### 3.2. Impact Factor Analysis on Self-Incompatibility Intensity of Differential Trait Samples

The distribution of genetic variation in the two sequencing samples is uneven, not only on different chromosomes in the same sample but also in different regions on the same chromosome. Similar results have also been found in other species [25,42,46]. We also analyzed the distribution of homozygous and heterozygous genetic variations in two sequencing samples. Due to the chromosomal recombination that occurs during the self-crossing process, the homozygous mutation rate and homozygous regions carried on each chromosome are different between the two sequencing samples. The differential distribution of homozygous regions on two samples may affect the green strawberry fruit-set rate, which suggests that homozygous regions are very likely to be where the self-incompatibility-related genes are located. Thus, differential genes between the two sequencing samples, especially the genes that hold most homozygous variations in one or two sequencing samples, deserve the most attention. Regarding why the growth rate of the pollen tubes from green strawberries is distinctly slower than that of *F. vesca* in their own style, we speculate that the mutations that occur in different lines in green strawberries compared to the reference *F. vesca* have changed the function of the related genes.

Ls-S_2_-53, Ls-S_2_-76, and *F. vesca* showed different fruit-set rates. Further study has supported the idea that the difference in fruit-set rate was caused by varying degrees of inhibition of the growth of pollen tubes in styles, which was derived from the fundamental sequence differences in the distribution of the sample. The accumulation of genetic variation or the formation of homozygotes after gene recombination will lead to the emergence of some new traits [25,53]. In order to study the effect of these mutations on sample traits, we calculated the distribution of nonsynonymous SNP mutations in the Pfam domain-containing genes, paying particular attention to the ratio of homozygous/heterozygous variations. In addition, we used the self-incompatibility-related genes involved in the mutations, with similar mutations to those that occurred in genes containing Pfam, to construct a linkage map, and found that the number of variations and homozygosity in the different genes was different (Additional File 8). In order to determine the function of the genes involved in the mutations corresponding to amino acid changes, we classified them by GO and KEGG analysis in order to allow us to better understand the relationship between genotype and mutation.

### 3.3. Analysis of the Potential Functions of Six Candidate Genes

ABC transporter can non-specifically bind S-RNase and transport extracellular S-RNase into pollen tubes to mediate a self-incompatibility reaction with the help of microfilaments and microtubules [54,55]. We detected three ABC transporters in which the expression profile of the *ABCA* gene was similar to MdABCF [54] and significantly associated with S-RNase transport. Calcium is the second messenger of cell signal transduction, and previous studies have confirmed that the Ca^2+^ gradient is involved in the self-incompatibility of pears [31,40]. We have detected three genes related to the formation of calcium ion gradients and its signal transduction. The gene *CDPK34*, a member of the calcium-dependent protein kinase family whose product can phosphorylate S-RNase [56], has similar expression patterns to *ABCA*, not only in the pistils but also in the anthers, and is presumably involved in the calcium signal transduction of the pollen tubes in the self-incompatibility reaction. Boron, as a trace element, is necessary for pollen germination and pollen tubes of fruit trees [57]. Although there is no relevant report on the involvement of boron in self-incompatibility, boron is known as a signaling molecule closely related to calcium gradients [58] and is involved in the formation of substances and intensity of pollen wall [59], and the improvement of fruit-set rate. In our results, we found two genes associated with boron transport, in particular, the *TIP5; 1* gene. It is speculated that the balance of intracellular and extracellular boron concentrations was broken after self-pollination, and further affects the normal growth of pollen tubes in the style. IOX is a key enzyme in UDP-glucuronic acid as a precursor of the plant cell wall [60] and ascorbate biosynthesis [61,62]. Large expression of *IOX2-like* after self-pollination may lead to a massive accumulation of cell wall material and the destruction of the reactive oxygen gradient of the pollen tube, which is similar to the phenomenon mediated by the self-incompatibility reaction. Methyl jasmonate small molecules may be involved in self-incompatibility reactions, with a different distribution in the style of self- and cross-pollination [33]. Phospholipase A1 is the first step in the biosynthesis of methyl jasmonate [63]. Increased expression levels of the gene (*PLA1IIgamma*) relating to its synthesis after pollination may be involved in the regulation of compatibility. FIP2 is a protein that regulates the actin skeleton [64,65]. Our result shows that the specific expression of *FIP2-like* in the pistil increased significantly after pollination, and the specific expression in the pollen may promote formation of the actin skeleton at the tip of the pollen tube, resulting in rapid growth of the pollen tube.

Analysis of expression patterns of self-incompatibility-modifying genes suggested that S-RNase was assisted by ABC family genes into pollen tubes. This may have triggered a variety of signal transduction systems to participate in self-incompatibility reaction in *F. viridis*. Previous research on incompatibility mechanisms in other species has found that relevant signals may mediate downstream events, such as microfilament depolymerization [39], the change of microstructures of cell walls and organelles [38,66], nuclear degradation [32,37], and programmed cell death [37], and eventually cause the pollen tube to stop growing within the style. Our results also suggest that if the functional changes of the modifier genes are involved in the self-incompatibility of *F. viridis*, the self-incompatibility intensity weakened, or enhanced, to varying degrees.

### 3.4. The Functional Verification of FIP2-Like

FIP2 is a protein that interacts with formin homology (FH), located in the membrane [64,67], that is a membrane localization protein, and both together may form a membrane-anchored complex that regulates the actin skeleton [64,65]. Our results show that FIP2-like can target the cell membrane, which supports the idea that FIP2-like performs a molecular function by associating with FH. The results also show that FIP2-like may act as a cofactor for actin cable formation and affect the germination of pollen grains and the growth of pollen tubes. Several plants possess an S-RNase-based SI system, and S-RNase acts as a style determinant into the pollen tube to degrade RNA and induce pollen tube death [29]. Moreover, pollen tubes quickly launch a self-protection mechanism to prevent S-RNase cytotoxicity [68]. The expression level of *FIP2-like* in pistil of the green strawberry increased significantly, however, there was no significant difference in *F. vesca* after manual self-pollination. S-RNase was specifically expressed in *F. viridis* pistil, however, interestingly, was not expressed in *F. vesca* species, which might be due to the loss of S-RNase in SC *Fragaria* [5]. S-RNase may enhance the expression of FIP2-like to slow down the process of F-actin degradation as a self-protection mechanism. Other evidence supports the lack of a significant difference in the expression level of *FIP2-like* between *F. vesca* (*F. vesca* 41) and *F. viridis* (Ls-S_2_-53) in germinated pollen tubes when both are mediated with no S-RNase. However, we found FIP2-like to have functional variations or loss in the green strawberries, which may be the reason for the different compatibility between Ls-S_2_-53 and Ls-S_2_-76.

The FIP2-like of *F. vesca* containing three domains (Figure 9c), the BTB domain, repeated pentapeptides, and the UBA domain, is from N- to C-terminal region, respectively. Repeated pentapeptide is a feature of FIP2 [64], the proteins containing the BTB domain can be involved in ubiquitin processes [69], and the UBA domain can bind to ubiquitinated proteins, which help to clear misfolded proteins [70]. FIP2-like may play an important role in promoting the correct assembly of F-actin. In addition to cytoskeletal regulation, another potential function of FIP2-like is transcriptional regulation. We speculate that shortened FIP2-like (P2) may participate in positive transcriptional regulation of the self-incompatibility reaction of Ls-S_2_-53, and cause pollen tubes to stop growing in the style. The functional evidence in transcriptional regulation is as follows: (1) The BTB domain is often found in transcription factors [71,72,73] whose functions are mainly governed by BTB domain-based protein–protein interactions [74,75,76]. (2) The protein containing the pentapeptide repeat was reported to interact with MED20, a subunit of the mediator complex, where it serves as a bridge between gene-specific regulatory proteins and the basal RNA polymerase II transcription machinery [71]. When repeated pentapeptides were lost and the BTB domain was damaged (P3), the ability of transcriptional regulation disappeared in Ls-S_2_-76 and the self-incompatibility intensity was correspondingly weakened.

## 4. Materials and Methods

### 4.1. Materials and Sample Selection

All of the plants were grown in the experimental field at the White Horse teaching and research base (Nanjing Agricultural University) in Jiangsu Province, China, from March to May 2017. Sample collection and pollination experiments were generally carried out between 08:30–10:00 when the weather was sunny, and the outside temperature was about 10 to 25 °C.

The fruit-set rate of *F. vesca* 41, *F. viridis* 42, Ls-S_1_-2, and 11 next-generation selfing lines of Ls-S_1_-2 were surveyed according to Ge et al. [6]. We selected young leaves of two second-generation selfing lines of *F. viridis* 42, Ls-S_2_-53 and Ls-S_2_-76, as materials for resequencing. Leaf, pedicel, calyx, petals, pistil, and anther samples of Ls-S_2_-53 were mainly collected for tissue-specific expression pattern analysis after manual self-pollination (0 h), and the pistils were collected after manual self-pollination (0, 6, 12, 24, 48, and 72 h) for gene spatiotemporal expression analysis. The flower organ samples used in the pollination experiment were all selected from the big budding stage [77].

### 4.2. Fruit-Set Rate Statistics and Dynamic Observation of Pollen Tube Growth

Anthers were collected, wrapped in air-laid paper, and then placed in a silicone bottle for drying for about 24 h at 4 °C. The dried pollen grains were directly used for isolation pollination or were stored in silicone bottles in a −70 °C freezer. The germination medium (GM) was modified according to Voyiatzis et al. [78] and Hiratsuka et al. [79], and contained 0.01% H_3_BO_3_, 0.01% KNO_3_, 0.01% MgSO_4_, 0.01% Ca(NO_3_)_2_·7H_2_O, 15% polyethylene glycol 4000 (PEG), 10% sucrose, and 0.5% MES, pH 6.0 ± 0.2. We determined the pollen grain germination rate before pollination and the active pollen grains were applied to the stigmas of stamen-emasculated flowers. We used double pollination (again, after six hours) and at least 50 flowers for the fruit-set statistics of each combination.

The styles after manual self-pollination (0, 6, 12, 24, 48, and 72 h) were fixed in FAA (5% formalin, 5% acetic acid, 65% ethanol) for two hours. The reader is referred to the method of Hiratsuka et al. [79] for staining. The styles were hydrolyzed in 4 M NaOH for two hours and stained in aniline blue staining solution (0.1% aniline blue, 0.1 M K_3_PO_4_) for 12 h after being washed three times. The pollen tube growth state in the styles was observed under a BX53 Olympus fluorescence microscope (Olympus, Tokyo, Japan). For each period, fifteen flowers were observed and, in each flower, 10 randomly selected styles were observed.

### 4.3. Resequencing and Mapping

Young leaves of Ls-S_2_-53 and Ls-S_2_-76 was used for genomic DNA extraction using a modified cetyltrimethylammonium bromide (CTAB) extraction protocol. DNA fragments of the desired length were gel-purified after being randomly sheared, and adapter ligation and DNA cluster preparation were performed, and sequencing was conducted using Solexa sequencing and an Illumina HiSeq 2500 (Illumina, San Diego, CA, USA). Low-quality reads (<20), reads with adaptor sequence, and duplicated reads were filtered, and the remaining high-quality data were used for mapping.

The clean sequencing reads were aligned to the *F. vesca* reference genome v2.0.a1 [9] using the Burrows–Wheeler Alignment tool v0.6.1 (Cambridge, UK) [80] under the default parameters with a small modification: allowing no more than three mismatches in the sequence and not allowing gaps (-o 0). The mapped reads were used to calculate average sequencing depth and coverage, and then detect SNP, indel, and SV polymorphisms.

### 4.4. Detection of SNPs, Indels, and SVs Polymorphisms

SAMtools software was used to eliminate redundancy (mark duplicates and PCR duplication) and selected read pairs with high mapping quality (>Q20) from the mapping result (Cambridge, UK) [81]. UnifedGenotyper in GATK (Cambridge, MA, USA) [82] was used to detect biallelic variants with high variant quality (>20) and indels were defined as the insertion or deletion, the length of which varied from 1 to 30 bp. SVs were generally described as large scale variations including insertion (INS), deletion (DEL), inversion (INV), ITX, and CTX. Firstly, we used Pindel (Cambridge, UK) [83] and Breakdancer (Washington University, St. Louis, MO, USA) [84] software to detect SVs with their default parameters. Secondly, in order to obtain reliable SVs, the detected SVs were returned to the paired reads alignments between sequencing samples and the reference, and were validated under the following criteria: 2× to 100× for coverage depth and more than 20 for SV quality.

### 4.5. Annotation of SNPs, Indels, and SVs

The annotation contained locations and functions were ascertained based on the information in GDR (The Genome Database for Rosaceae). SnpEff software (Wayne State University, Detroit, MI, USA) [85] was used to annotate SNPs and small indels, and polymorphisms were annotated as genic and intergenic, depending on whether they were in the gene region. The SNPs, indels, and SVs were classified as exonic, intronic, etc., according to the specific location in the genic region. SNPs in the exons of CDS were further classified according to the variation of the amino acid residues it caused, such as synonymous or nonsynonymous. In order to get annotation for analyzing gene function, we blasted [86] each gene containing mutations to NR [87], Swiss-Prot [87], GO [88], COG [89], and KEGG [90] database.

### 4.6. Search and Prediction of Genes Related to Self-Incompatibility

Gametophytic self-incompatibility can inhibit the normal growth of germinated isogenic pollen tubes in the style as a complex defense mechanism. The specificity of the GSI interaction is determined by S-locus genes; however, the presence of genes at other loci (modifiers) is required for full manifestation of the GSI response [29]. These modifiers, together with S-RNase, undertake the regulation of physiological and biochemical metabolism and signal cascade responses of pollen tubes in styles [29]. We chose related genes mainly based on previous research in other gametophyte self-incompatibility species. Prediction of candidate effectors was performed according to the change of a gene’s function between two sequencing simple genomes and the reference genome. In brief, genes in which SNPs occurred, including nonsynonymous, Stopgain, Stop_Lost, etc.; indels including Fram- shift, Codon_Deletion, etc.; and SVs in coding sequence were chosen as candidate genes. A total of seventeen genes were selected for further tissue-specific analysis by testing their expression levels in pistil after manual self-pollination.

### 4.7. RT-qPCR

Total RNA of strawberry tissue was extracted with the plant total RNA isolation Kit Plus from Fuji (Chengdu, China) according to the manufacturer’s protocol. The concentration of total RNA was measured using a NanoDrop 2000 UV-Vis spectrophotometer (Thermo Scientific, Waltham, MA, USA) after treatment of genomic DNA with RNase-free DNase I (Takara, Dalian, China). PrimeScript^TM^ RT reagent Kit (Takara) was used to obtain cDNA according to the manufacturer’s instructions. Synthesized cDNA concentration was diluted to 100 ng/µL and each reaction mixture contained 10.0 µL SYBR Premix Ex Taq^TM^ (Takara), 0.5 µL of each primer (10 µM), 1 µL cDNA, and 8 µL ddH_2_O in a total volume of 20 µL. Reactions were performed under the following conditions: 95 °C preheating for 4 min, followed by 40 cycles at 95 °C for 20 s, 60 °C for 20 s, and 72 °C for 40 s. An elongation factor-α gene (EF1-α) was used as a standard control in the RT-qPCRs. The primer pairs were designed in the CDS area of *F. vesca.* The forward and reverse primers are shown in Table S1. All primers were subjected to normal PCR and the reaction products were separated on a 1% agarose gel to ensure the bands were of the expected size and that there were no primer dimers. The PCR experiment was carried out with at least three technical replicates. The relative transcript levels of selected genes were calculated using the 2^−ΔΔ*C*t^ method [91].

### 4.8. Cloning and Sequence Analysis of FIP2-like

The open reading frame (ORF) of the *FIP2-like* gene was obtained using the specific primers FIP2-like-ORF (Table S1) and PCR using PrimeSTAR^®^ GXL DNA polymerase (Takara), which was also used to compare sequences between different samples. The amplification procedure was as follows: 98 °C for 5 min; 36 cycles at 98 °C for 10 s, 53 °C for 15 s, and 68 °C for 1 min; and a final extension at 68 °C for 10 min. The PCR products were analyzed on 1.0% agarose gels, and the putative fragment was purified using a DNA purification kit (Takara). The amplified fragment was cloned into the pMDTM 19-T Vector (Takara), transformed into *Escherichia coli* DH5α competent cells (Tsingke, Nanjing, China), and 10 monoclones per sample were sequenced (Tsingke). The nucleotide sequence and transcriptional amino acid sequence of *FIP2-like* were analyzed using alignment tool DNAMAN8.0 (Lynnon, QC, Canada). The domain of FIP2-like was predicted using the CDD Tool from the NCBI databases.

### 4.9. Visualization of F-Actin Organization In Vitro

Actin filaments in fixed pollen tubes were stained with Alexa Fluor 488 phalloidin (Invitrogen, Carlsbad, CA, USA) according to Jia et al. [92]. The pollen grains germinated in GM for 3 h were fixed and washed according to the following steps: first, pollen tubes were fixed in a freshly prepared solution of 4% paraformaldehyde in PBS (pH 6.8) for 1.5 h, and then gently washed with PBS (pH 6.8) twice, and then in enzyme solution of 1% cellulase R-10 and 1% pectinase for 20 min. Pollen tubes were incubated in 1% Triton X-100 to increase cell membrane permeability, and then once in PBS. Finally, the actin cytoskeleton was stained with 0.33 µM Alexa 488-phalloidin followed by three washes with PBS. The samples were mounted onto glass slides and photographed for further analysis of F-actin intensity using a Zeiss LSM 800 confocal laser scanning microscope (LSCM) (Zeiss, Jena, Germany) with excitation at 488 nm. At least 50 tubes were analyzed for each treatment, which was repeated three times.

### 4.10. Subcellular Localization of FIP2-Like

For *FIP2-like* (geneID for gene01424) subcellular localization vector constructs, full-length sequences were amplified from *F. vesca* 41 anther cDNA and directionally cloned into entry vector pDONR221 using specific primers FIP2-like-Sub (Table S1), and the resulting *FIP2-like* gene fragment was subsequently subcloned into Gateway ready pMDC43 vector, using the LR reaction of Gateway recombination-based cloning (Invitrogen). The constructed fusion vector was transferred into *Agrobacterium* and used to transform young *N. benthamiana* leaves. The FIP2-like-GFP protein fluorescence was observed using a Zeiss LSM 800 (Zeiss) 2–3 days after transformation.

### 4.11. Transient Expression Assay

Phosphorothioated as-ODN treatment was performed essentially as described by Meng et al. [54]. We designed an as-ODN based on the CDS region of *FIP2-like* described, to downregulate *FIP2-like*. First, taking a small amount of pollen (20 pollen chambers) into a 1.5 mL GM in a centrifuge tube, the culture was shaken at 200 rpm at 25 °C in the darkness. In addition to adding 210 µL cytofectin buffer and 30 µL cytofectin to the medium, we also added either as-ODN (1 µM final concentration), or s-ODN as controls, and incubated for 3 h. We then took 100 µL liquid medium containing pollen tubes for germination rate measurements and pollen tube length measurements, and 100 µL liquid to observe actin analysis; three biological replicates were made, using at least 50 pollen grains. The remaining pollen grains were collected by centrifugation at 6000× *g* and 6 min, for use in RT-qPCR analysis.

## 5. Conclusions

In this study, we sequenced two selfing lines of *F. viridis* and an *F. vesca* reference. A large number of variations (SNPs, indels, and SVs) were obtained and annotated among three differential trait samples, which provided basic research data of *Fragaria* for functional genomic analyses, molecular breeding, molecular markers, genetic evolutionary relationship, and more. We further analyzed the distribution patterns of the variations and found that the distribution region and the number of the homozygous variations were especially different on the chromosomes of the two sequencing samples, which could explain the different fruit-set rate or self-incompatibility intensities. We screened and functionally annotated the sequence variations that might lead to changes in the encoded amino acids between the three different samples, and predicted the identity of the gene related to self-incompatibility of *F. viridis*. Seventeen candidate genes were selected for tissue-specific expression pattern analysis, and it was found that these genes were abundantly expressed in floral organs, especially in anthers and styles. The spatiotemporal specificity analysis of gene expression revealed that six genes were significantly associated with self-incompatibility. The gene *FIP2-like* was focused upon; this gene is associated with actin cytoskeleton formation, and its translation process was terminated prematurely due to FRAME_SHIFT at different sites in two selfing lines with *F. vesca* as the reference. Our experimental evidence suggests that the fully encoded FIP2-like from *F. vesca* can function normally and promote F-actin formation. The shortened amino acid sequences may have new features and have a positive transcription regulation role in the self-incompatibility reaction. Although the difference between the three sample traits can be explained by the amino acid composition of FIP2-like, the function of the shortened sequences needs to be further confirmed. Additionally, there are many factors involved in self-incompatibility intensity, and the difference in fruit-set rate may be coordinated by several modifiers, which also need to be further explored.

## Figures and Tables

**Table 1:** Fruit-set rate of *Fragaria viridis* and self-cross accessions.

Lines	*F. vesca* 41	Ls	Ls-S_1_-2	Ls-S_2_-02	Ls-S_2_-11	Ls-S_2_-28	Ls-S_2_-30	Ls-S_2_-37	Ls-S_2_-49	Ls-S_2_-53	Ls-S_2_-57	Ls-S_2_-61	Ls-S_2_-63	Ls-S_2_-76
Fruit-set rate	100%	20%	25%	30%	40%	18%	80%	11%	16%	2%	12%	18%	12%	75%
Aberration rate	0%	100%	100%	100%	68%	86%	60%	67%	86%	100%	100%	100%	83%	32%

Ls represents *F. viridis* 42, the zeroth generation, which is taken as reference for the assessment of the fruit-set and the (in)compatibility. S_1_ and S_2_ represent the first and second self-crossing generations, respectively. Aberration rate indicates the ratio of stunted fruits (with few achenes and irregular shapes) to the total number of fruits.

**Table 2:** The proportion of styles traversed by at least one pollen tube.

Lines	*F. vesca* 41	Ls-S_2_-53	Ls-S_2_-76
0 h	0% (0/150)		
6 h			
12 h			
24 h	80% (120/150)	0% (0/150)	12% (18/150)
48 h	100% (150/150)	6% (9/150)	90% (135/150)
72 h	100% (150/150)	6% (9/150)	89% (134/150)

The numerators in parentheses represent the number of styles crossed by pollen tube and the denominators represent all the styles observed.

**Table 3:** Statistical analysis of resequencing data for *F. viridis* sequencing samples Ls-S_2_-76 and Ls-S_2_-53.

Sample	Total_Reads	Clean_Reads	Clean-Base	Q30 (%)	Mapped (%)	Depth	GC (%)	Cov_Ratio_1× (%)	Duplication (%)	Mean Mapping Quality
Ls-S_2_-53	72,849,376	63,518,251	19,055,475,300	91.34	91.64	80.379	39.17	90.64	33.24%	52.0563
Ls-S_2_-76	66,877,412	58,811,565	17,643,469,500	91.20	92.48	75.067	39.00	90.60	31.72%	52.0845

**Table 4:** Variations of *FIP2-like* in *F. vesca* 41, Ls-S_2_-76, and Ls-S_2_-53.

Gene	Variation Type	Location	*F. vesca* 41	Ls-S_2_-53	Ls-S_2_-76
*FIP2-like*	Syn	29162859	A	G	G
	Syn	29162874	C	T	T
	FRAME_SHIFT	29162967	CAC	CAC, A	A
	Syn	29163022	G	G, T	T
	Nonsyn	29163188	T	C	C
	Syn	29163231	T	C	C
	Nonsyn	29163281	A	G	G
	Nonsyn	29163300	G	T	T
	Syn	29163420	C	T	T
	Syn	29163461	A	A, C	C
	Nonsyn	29163566	A	C	C
	Syn	29163618	G	A	A
	Syn	29163639	C	T	T
	Nonsyn	29163668	T	T, C	T
	FRAME_SHIFT	29163671	ACTGTAAATTTGTC AAGTCAAATATCC GGGGA	AATGTAAATTTGTC AAGTCAAATATCC GGGGA, A	AATGTAAATTTGTC AAGTCAAATATCC GGGGA
	Syn	29163735	G	G, A	G
	Syn	29163764	G	A	A
	Syn	29163836	C	T	T

## Supplementary Materials

Supplementary materials can be found at https://www.mdpi.com/1422-0067/20/5/1039/s1.

## Abbreviations

SNP	Single-nucleotide polymorphism
Indel	Insertion and deletion
SV	Structure Variation
GSI	gametophytic self-incompatibility
SI	self-incompatibility
SC	self-compatibility
PCD	programmed cell death
s-ODN	sense oligodeoxynucleotides
as-ODN	antisense oligodeoxynucleotides
GM	germination medium
PBS	phosphate buffer saline
NR	NCBI non-redundant protein
CGO	cluster of ortholog genes
GO	gene ontology
KEGG	Kyoto encyclopedia of genes and genomes
RT-qPCR	Reverse transcription and quantitative real-time PCR
FIP	FH protein interacting protein
ABC	ATP-binding cassette
CDPK	calcium-dependent protein kinase
TIP	tonoplast membrane intrinsic protein
IOX	inositol oxygenase
PLA1IIgamma	phospholipase A1-IIgamma
FH	Formin Homology

## References

[1] Sargent D.J., Geibel M., Hawkins J.A., Wilkinson M.J., Battey N.H., Simpson D.W. (2004). Quantitative and qualitative differences in morphological traits revealed between diploid *Fragaria* species. Ann. Bot..

[2] Staudt G. (1952). Cytogenetische untersuchungen an *Fragaria* orientalis, Los. und ihre Bedeutung Für Artbildung und Geschlechtsdifferenzierung in der Gattung *Fragaria*, L. Zeitschrift Induktive Abstammungs Vererbungslehre.

[3] Evans W.D., Jones J.K. (1967). Incompatibility of *Fragaria*. Can. J. Genet. Cytol..

[4] Sassa H. (2016). Molecular mechanism of the S-RNase-based gametophytic self-incompatibility in fruit trees of Rosaceae. Breed. Sci..

[5] Bošković R.I., Sargent D.J., Tobutt K.R. (2010). Genetic evidence that two independent S-loci control RNase- based self-incompatibility in diploid strawberry. J. Exp. Bot..

[6] Ge C.F., Zhang H.Y., Chen B.Y., Cai B.H., Qiao Y.S. (2014). Characteristics and evaluation of self- ross progenies of *Fragaria viridis* Duch. J. Plant Genet. Resour..

[7] Velasco R., Zharkikh A., Affourtit J., Dhingra A., Cestaro A., Kalyanaraman A., Fontana P., Bhatnagar S.K., Troggio M., Pruss D. (2010). The genome of the domesticated apple (*Malus × domestica* Borkh.). Nat. Genet..

[8] Wu J., Wang Z.W., Shi Z.B., Zhang S., Ming R., Zhu S.L., Khan M.A., Tao S.T., Korban S.S., Wang H. (2013). The genome of the pear (*Pyrus bretschneideri* Rehd.). Genome Res..

[9] Shulaev V., Sargent D.J., Crowhurst R.N., Mockler T.C., Folkerts O., Delcher A.L., Jaiswal P., Mockaitis K., Liston A., Mane S.P. (2010). The genome of woodland strawberry (*Fragaria vesca*). Nat. Genet..

[10] Verde I., Abbott A.G., Scalabrin S., Jung S., Shu S., Marroni F., Zhebentyayeva T., Dettori M.T., Grimwood J., Cattonaro F. (2013). The high-quality draft genome of peach (*Prunus persica*) identifies unique patterns of genetic diversity, domestication and genome evolution. Nat. Genet..

[11] Zhang Q.X., Chen W.B., Sun L.D., Zhao F.Y., Huang B.Q., Yang W.R., Tao Y., Wang J., Yuan Z.Q., Fan G.Y. (2012). The genome of *Prunus mume*. Nat. Commun..

[12] Tennessen J.A., Govindarajulu R., Liston A., Ashman T.L. (2013). Targeted sequence capture provides insight into genome structure and genetics of male sterility in a gynodioecious diploid strawberry, *Fragaria vesca* ssp. *bracteata* (Rosaceae). G3 Genes, Genomes Genet..

[13] Darwish O., Shahan R., Liu Z., Slovin J.P., Alkharouf N.W. (2015). Re-annotation of the woodland strawberry (*Fragaria vesca*) genome. BMC Genom..

[14] Edger P.P., VanBuren R., Colle M., Poorten T.J., Wai C.M., Niederhuth C.E., Alger E.I., Ou S.J., Acharya C.B., Wang J. (2017). Single-molecule sequencing and optical mapping yields an improved genome of woodland strawberry (*Fragaria vesca*) with chromosome-scale contiguity. GigaScience.

[15] Li Y., Wei W., Feng J., Luo H., Pi M., Liu Z., Kang C. (2017). Genome re-annotation of the wild strawberry *Fragaria vesca* using extensive Illumina-and SMRT-based RNA-seq datasets. DNA Res..

[16] Tennessen J.A., Govindarajulu R., Ashman T.L., Liston A. (2014). Evolutionary origins and dynamics of octoploid strawberry subgenomes revealed by dense targeted capture linkage maps. Genome Biol. Evol..

[17] Hollender C.A., Kang C., Darwish O., Geretz A., Matthews B.F., Slovin J., Alkharouf N., Liu Z.C. (2014). Floral transcriptomes in woodland strawberry uncover developing receptacle and anther gene networks. Plant Physiol..

[18] Sargent D.J., Passey T., Šurbanovski N., Lopez G.E., Kuchta P., Davik J., Harrison R., Passey A., Whitehouse A.B., Simpson D.W. (2012). A microsatellite linkage map for the cultivated strawberry (*Fragaria* x ananassa) suggests extensive regions of homozygosity in the genome that may have resulted from breeding and selection. Theor. Appl. Genet..

[19] Bassil N.V., Davis T.M., Zhang H., Ficklin S., Mittmann M., Webster T., Mahoney L., Wood D., Alperin E.S., Rosyara U.R. (2015). Development and preliminary evaluation of a 90 K Axiom^®^ SNP array for the allo-octoploid cultivated strawberry *Fragaria*× *ananassa*. BMC Genom..

[20] Cao K., Zhou Z., Wang Q., Guo J., Zhao P., Zhu G.R., Fang W.C., Chen C.W., Wang X.W., Wang X.L. (2016). Genome-wide association study of 12 agronomic traits in peach. Nat. Commun..

[21] Kunihisa M., Moriya S., Abe K., Okada K., Haji T., Hayashi T., Kawahara Y., Itoh R., Itoh T., Katayose Y. (2016). Genomic dissection of a ‘Fuji’ apple cultivar: Re-sequencing, SNP marker development, definition of haplotypes, and QTL detection. Breed. Sci..

[22] Lee H.S., Kim G.H., Kwon S.I., Kim J.H., Kwon Y.S., Choi C. (2016). Analysis of ‘Fuji’ apple somatic variants from next-generation sequencing. Genet. Mol. Res..

[23] Wang L., Peng Q., Zhao J., Ren F., Zhou H., Wang W., Liao L., Owitti A., Jiang Q., Han Y.P. (2016). Evolutionary origin of Rosaceae-specific active non-autonomous hAT elements and their contribution to gene regulation and genomic structural variation. Plant Mol. Biol..

[24] Cao K., Zheng Z., Wang L., Liu X., Zhu G.G., Fang W.C., Cheng S.F., Zeng P., Chen C.W., Wang X.W. (2014). Comparative population genomics reveals the domestication history of the peach, *Prunus persica*, and human influences on perennial fruit crops. Genome Biol..

[25] Xing L., Zhang D., Song X., Weng K., Shen Y.W., Li Y.M., Zhao C.P., Ma J.J., An N., Han M.Y. (2016). Genome-wide sequence variation identification and floral-associated trait comparisons based on the re-sequencing of the ‘Nagafu No. 2’and ‘Qinguan’varieties of apple (*Malus domestica* Borkh.). Front. Plant Sci..

[26] Xu Y.S., Gao Z.H., Tao J.M., Jiang W.H., Zhang S.J., Wang Q.N., Qu S.C. (2016). Genome-wide detection of SNP and SV variations to reveal early ripening-related genes in grape. PLoS ONE.

[27] Samad S., Kurokura T., Koskela E., Toivainen T., Patel V., Mouhu K., Sargent D.J., Hytönen T. (2017). Additive QTLs on three chromosomes control flowering time in woodland strawberry (*Fragaria vesca* L.). Hortic. Res..

[28] Franklin-Tong N.V.E., Franklin F.C.H. (2003). Gametophytic self-incompatibility inhibits pollen tube growth using different mechanisms. Trends Plant Sci..

[29] Wu J., Gu C., Khan M.A., Wu J.Y., Gao Y.B., Wang C.L., Korban S.S., Zhang S.L. (2013). Molecular determinants and mechanisms of gametophytic self-incompatibility in fruit trees of Rosaceae. Crit. Rev. Plant Sci..

[30] Gu Z., Meng D., Yang Q., Yuan H., Wang A., Li W., Chen Q.J., Zhang Y., Wang D.M., Li T.Z. (2015). A CBL gene, *MdCBL5*, controls the calcium signal and influences pollen tube growth in apple. Tree Genet. Genom..

[31] Qu H., Zhang Z., Wu F., Wang Y. (2016). The role of Ca^2+^ and Ca^2+^ channels in the gametophytic self-incompatibility of *Pyrus pyrifolia*. Cell Calcium.

[32] Wang C.L., Wu J., Xu G.H., Gao Y.B., Chen G., Wu J.Y., Wu H.Q., Zhang S.L. (2010). S-RNase disrupts tip-localized reactive oxygen species and induces nuclear DNA degradation in incompatible pollen tubes of *Pyrus pyrifolia*. J. Cell Sci..

[33] Shi D., Tang C., Wang R., Gu C., Wu X., Hu S., Jiao J., Zhang S.L. (2017). Transcriptome and phytohormone analysis reveals a comprehensive phytohormone and pathogen defence response in pear self-/cross-pollination. Plant Cell Rep..

[34] Lee C.B., Kim S., McClure B. (2009). A pollen protein, NaPCCP, that binds pistil arabinogalactan proteins also binds phosphatidylinositol 3-phosphate and associates with the pollen tube endomembrane system. Plant Physiol..

[35] Qu H., Guan Y., Wang Y., Zhang S.L. (2017). PLC-mediated signaling pathway in pollen tubes regulates the gametophytic self-incompatibility of *pyrus* species. Front. Plant Sci..

[36] Li W., Meng D., Gu Z., Qing Y., Hui Y., Yang L., Chen Q.J., Yu J., Liu C.S., Li T.Z. (2018). Apple S-RNase triggers inhibition of tRNA aminoacylation by interacting with a soluble inorganic pyrophosphatase in growing self-pollen tubes in vitro. New Phytol..

[37] Wang C.L., Xu G.H., Jiang X.T., Chen G., Wu J., Wu H.Q., Zhang S.L. (2009). S-RNase triggers mitochondrial alteration and DNA degradation in the incompatible pollen tube of *Pyrus pyrifolia* in vitro. Plant J..

[38] Graaf B.H., Knuiman B.A., Derksen J., Mariani C. (2003). Characterization and localization of the transmitting tissue-specific PELPIII proteins of *Nicotiana tabacum*. J. Exp. Bot..

[39] Liu Z., Xu G., Zhang S. (2007). Pyrus pyrifolia stylar S-RNase induces alterations in the actin cytoskeleton in self-pollen and tubes in vitro. Protoplasma.

[40] Xu G., Zhang S., Yang Y., Zhao C.P., Wolukau J.N. (2008). Influence of endogenous and exogenous RNases on the variation of pollen cytosolic-free Ca^2+^ in *Pyrus serotina Rehd*. Acta Physiologiae Plantarum.

[41] Matsumoto D., Tao R. (2012). Isolation of pollen-expressed actin as a candidate protein interacting with S-RNase in *Prunus avium* L. J. Jpn.Soc. Hortic. Sci..

[42] Bai H., Cao Y., Quan J., Dong L., Li Z.Y., Zhu Y.B., Zhu L.H., Dong Z.P., Li D.Y. (2013). Identifying the genome-wide sequence variations and developing new molecular markers for genetics research by re-sequencing a landrace cultivar of foxtail millet. PLoS ONE.

[43] Ge C.F., Du J.K., Xiong J.S., Wang S.H., Qiao Y.S., Wang C.Y. (2018). Integrative Analysis of Transcriptome and Proteome Provides Comprehensive Insights into the Mechanism of *Fragaria viridis* Self-Incompatibility.

[44] Lam H.M., Xu X., Liu X., Chen W., Yang G., Wong F., Li M., He W., Qin N., Wang B. (2010). Resequencing of 31 wild and cultivated soybean genomes identifies patterns of genetic diversity and selection. Nat. Genet..

[45] Xu X., Liu X., Ge S., Jensen J.D., Hu F.Y., Li X., Dong Y., Gutenkunst R.N., Fang L., Huang L. (2012). Resequencing 50 accessions of cultivated and wild rice yields markers for identifying agronomically important genes. Nat. Biotechnol..

[46] Jiang S.K., Sun S.C., Bai L.M., Ding G.H., Wang T.T., Xia T.S., Jiang H., Zhang X.J., Zhang F.M. (2017). Resequencing and variation identification of whole genome of the japonica rice variety ‘Longdao24’ with high yield. PLoS ONE.

[47] Lei J.J., Xue L., Dai H.P., Deng M.Q., Zhang Y.T., Lei J.J. (2015). Study on Taxonomy of the Strawberry Genus (Fragaria) in the Word. Advances in Strawberry Research (IV).

[48] Staudt G., DiMeglio L.M., Davis T.M., Gerstberger P. (2003). *Fragaria* × *bifera* Duch.: Origin and taxonomy. Botanische Jahrbücher Systematik Pflanzengeschichte Pflanzengeographie.

[49] Lin J., Davis T.M. (2000). S1 analysis of long PCR heteroduplexes: Detection of chloroplast indel polymorphisms in *Fragaria*. Theor. Appl. Genet..

[50] Hazzouri K.M., Flowers J.M., Visser H.J., Khierallah H.S.M., Rosas U., Pham G.M., Meyer R.S., Johansen C.K., Fresquez Z.A., Masmoudi K. (2015). Whole genome re-sequencing of date palms yields insights into diversification of a fruit tree crop. Nat. Commun..

[51] Lee W., Jiang Z., Liu J., Haverty P.M., Guan Y.H., Stinson J.M., Yue P., Zhang Y., Pant K.P., Bhatt D. (2010). The mutation spectrum revealed by paired genome sequences from a lung cancer patient. Nature.

[52] Pleasance E.D., Cheetham R.K., Stephens P.J., McBride D.J., Humphray S.J., Greenman C.D., Varela. I., Lin M.L., Ordóñez G.R., Bignell G.R. (2010). A comprehensive catalogue of somatic mutations from a human cancer genome. Nature.

[53] Slovin J.P., Kyle S., Folta K.M. (2009). An inbred line of the diploid strawberry *Fragaria vesca* f.semperflorensfor genomic and molecular genetic studies in the Rosaceae. Plant Methods.

[54] Meng D., Gu Z., Li W., Wang A., Yuan H., Yang Q., Li T.Z. (2014). Apple MdABCF assists in the transportation of S-RNase into pollen tubes. Plant J..

[55] Meng D., Gu Z., Yuan H., Wang A., Li W., Yang Q., Zhu Y.D., Li T.Z. (2014). The microtubule cytoskeleton and pollen tube Golgi vesicle system are required for in vitro S-RNase internalization and gametic self-incompatibility in apple. Plant Cell Physiol..

[56] Kunz C., Chang A., Faure J.D., Clarke A.E., Polya G.M., Anderson M.A. (1996). Phosphorylation of style S-RNases by Ca^2+^-dependent protein kinases from pollen tubes. Sex. Plant Reprod..

[57] Kizildemir M., Bolat I., Ak B.E. Effects of foliar boron applications to almond on some pollen features and fruit set. Proceedings of the VII International Symposium on Mineral Nutrition of Fruit Crops.

[58] Fang K., Zhang W., Xing Y.Q., Yang L., Cao Q.Q., Qin L. (2016). Boron toxicity causes multiple effects on *Malus domestica* pollen tube growth. Front. Plant Sci..

[59] Tanaka N., Uraguchi S., Saito A., Kajikawa M., Kasai K., Sato Y., Nagamura Y., Fujiwara T. (2013). Roles of pollen-specific boron efflux transporter, OsBOR4, in the rice fertilization process. Plant Cell Physiol..

[60] Kanter U., Usadel B., Guerineau F., Li Y., Pauly M., Tenhaken R. (2005). The inositol oxygenase gene family of Arabidopsis is involved in the biosynthesis of nucleotide sugar precursors for cell-wall matrix polysaccharides. Planta.

[61] Lorence A., Chevone B.I., Mendes P., Nessler C.L. (2004). Myo-inositol oxygenase offers a possible entry point into plant ascorbate biosynthesis. Plant Physiol..

[62] Sharma P., Dubey R.S. (2005). Drought induces oxidative stress and enhances the activities of antioxidant enzymes in growing rice seedlings. Plant Growth Regul..

[63] Hatakeyama K., Ishiguro S., Okada K., Takasaki T., Hinata K. (2003). Antisense inhibition of a nuclear gene, *BrDAD1*, in Brassica causes male sterility that is restorable with jasmonic acid treatment. Mol. Breed..

[64] Banno H., Chua N.H. (2000). Characterization of the Arabidopsis formin-like protein AFH1 and its interacting protein. Plant Cell Physiol..

[65] Soellick T.R., Uhrig J.F. (2001). Development of an optimized interaction-mating protocol for large-scale yeast two-hybrid analyses. Genome Biol..

[66] Herrero M., Dickinson H.G. (1981). Pollen tube development in Petunia hybrida following compatible and incompatible intraspecific matings. J. Cell Sci..

[67] Cheung A.Y., Wu H. (2004). Overexpression of an Arabidopsis formin stimulates supernumerary actin cable formation from pollen tube cell membrane. Plant Cell.

[68] Chen J., Wang P., De B.G., Zhang H.H.J., Tang C., Zhang S.L., Wu J.Y. (2018). Phosphatidic acid counteracts S-RNase signaling in pollen by stabilizing the actin cytoskeleton. Plant Cell.

[69] Li J.H., Su X.X., Wang Y.L., Yang W., Pan Y., Su C.G., Zhang X.G. (2018). Genome-wide identification and expression analysis of the BTB domain-containing protein gene family in tomato. Genes Genom..

[70] Cabe M., Rademacher D.J., Karlsson A.B., Cherukuri S., Bakowska J.C. (2018). PB1 and UBA domains of p62 are essential for aggresome-like induced structure formation. Biochem. Biophys. Res. Commun..

[71] Skoblov M., Marakhonov A., Marakasova E., Guskova A., Chandhoke V., Birerdinc A., Baranova A. (2013). Protein partners of KCTD proteins provide insights about their functional roles in cell differentiation and vertebrate development. Bioessays.

[72] Davies E.L., Lim J.G.Y., Joo W.J., Tam C.H., Fuller M.T. (2013). The transcriptional regulator lola is required for stem cell maintenance and germ cell differentiation in the *Drosophila* testis. Dev. Biol..

[73] Melnick A., Ahmad K.F., Arai S., Polinger A., Ball H., Borden K.L., Carlile G.W., Prive G.G., Licht J.D. (2000). In-depth mutational analysis of the promyelocytic leukemia zinc finger BTB/POZ domain reveals motifs and residues required for biological and transcriptional functions. Mol. Cell Biol..

[74] Melnick A., Carlile G., Ahmad K.F., Kiang C.L., Corcoran C., Bardwell V., Prive G.G., Licht J.D. (2002). Critical residues within the BTB domain of PLZF and Bcl-6 modulate interaction with corepressors. Mol. Cell. Biol..

[75] Bonchuk A., Denisov S., Georgiev P., Maksimenko O. (2011). Drosophila BTB/POZ domains of “ttk group” can form multimers and selectively interact with each other. J. Mol. Biol..

[76] Weber H., Bernhardt A., Dieterle M., Hano P., Mutlu A., Estelle M., Genschik P., Hellmann H. (2005). Arabidopsis AtCUL3a and AtCUL3b form complexes with members of the BTB/POZ-MATH protein family. Plant Physiol..

[77] Wang Z.W., Zhao M.Z., Qiao Y.M., Wu W.M., Yuan J. (2010). Study on pollen viability of strawberry. J. Northeast Agric. Univ..

[78] Voyiatzis D.G., Paraskevopoulou-Paroussi G. (2002). Factors affecting the quality and in vitro germination capacity of strawberry pollen. J. Hortic. Sci. Biotechnol..

[79] Hiratsuka S., Zhang S.L., Nakagawa E., Kawai W. (2001). Selective inhibition of the growth of incompatible pollen tubes by S-protein in the Japanese pear. Sex. Plant Reprod..

[80] Li H., Durbin R. (2009). Fast and Accurate Short Read Alignment with Burrows–Wheeler Transform.

[81] Li H., Handsaker B., Wysoker A., Fennell T., Ruan J., Homer N., Gabor N., Marth G., Abecasis G., Durbin R. (2009). The sequence Alignment/Map (SAM) format and SAMtools. Transpl. Proc..

[82] McKenna A., Hanna M., Banks E., Sivachenko A., Cibulskis K., Kernytsky A., Garimella K., Altshuler D., Gabriel S., Daly M. (2010). The genome analysis toolkit: A MapReduce framework for analyzing next-generation DNA sequencing data. Genome Res..

[83] Ye K., Schulz M.H., Long Q., Apweiler R., Ning Z., Notes A. (2009). Pindel: A pattern growth approach to detect break points of large deletions and medium sized insertions from paired-end short reads. Bioinformatics.

[84] Chen K., Wallis J.W., Mclellan M.D., Larson D.E., Kalicki J.M., Pohl C.S., McGrath S.D., Wendl M.C., Zhang Q.Y., Locke D.P. (2009). BreakDancer: An algorithm for high-resolution mapping of genomic structural variation. Nat. Methods.

[85] Cingolani P., Platts A., Wang L.L., Coon M., Nguyen T., Wang L., Land S.J., Lu X.Y., Ruden D.M. (2012). A program for annotating and predicting the effects of single nucleotide polymorphisms. Fly.

[86] Altschul S.F., Madden T.L. (1997). A new generation of protein database search programs. Nucleic Acids Res..

[87] Deng Y.Y., Li J.Q., Wu S.F., Zhu Y.P., Chen Y.W., He F.C. (2006). Integrated nr database in protein annotation system and its localization. Comput. Eng..

[88] Ashburner M., Ball C.A., Blake J.A., Botstein D., Butler H., Cherry J.M., Davis A.P., Dolinski K., Dwight S.S., Eppig J.T. (2000). Gene ontology: Tool for the unification of biology. Nat. Genet..

[89] Tatusov R.L., Galperin M.Y., Natale D.A., Koonin E.V. (2000). The COG database: A tool for genome-scale analysis of protein functions and evolution. Nucleic Acids Res..

[90] Kanehisa M., Goto S., Kawashima S., Okuno Y., Hattori M. (2004). The KEGG resource for deciphering the genome. Nucleic Acids Res..

[91] Livak K.J., Schmittgen T.D. (2001). Analysis of relative gene expression data using real-time quantitative PCR and the 2^−ΔΔCT^ method. Methods.

[92] Jia H., Yang J., Liesche J., Liu X.,  Hu Y., Si W.,  Guo J., Li J. (2017). Ethylene promotes pollen tube growth by affecting actin filament organization via the cGMP-dependent pathway in *Arabidopsis thaliana*. Protoplasma.

